# Wege zum erfolgreichen Mentoring in der Radiologie

**DOI:** 10.1007/s00117-022-01030-6

**Published:** 2022-07-19

**Authors:** Benjamin Sigl, Christian Herold

**Affiliations:** Universitätsklinik für Radiologie und Nuklearmedizin, Medizinische Universität Wien, 1090 Wien, Österreich

## Darum geht es:

✓ Welche Arten von Mentoring gibt es?

✓ Was macht einen guten Mentor/Mentee aus?

✓ Wie kann eine Institution zum Erfolg eines Mentoring-Programms beitragen?

## Warum Mentoring?

Mit zunehmender Relevanz der Radiologie in der modernen Medizin und damit einhergehenden steigenden Untersuchungszahlen, zunehmender Komplexität von Untersuchungen und vermehrten administrativen Tätigkeiten steigt auch der Druck auf den radiologischen Nachwuchs. Untersuchungen haben insbesondere zu Beginn und im mittleren Abschnitt der Assistenzarztausbildung ein erhöhtes Stresspotenzial und damit einhergehend steigende Abbruchraten, Burnout-Phänomene, Depression und Versagensängste gezeigt [1–3]. Erfolgreiche Mentoring-Programme in der Radiologie tragen nicht nur zu einem reibungslosen Einstieg in das Berufsleben bei, sondern können auch nachhaltig die Karriere junger Radiologinnen und Radiologen positiv beeinflussen, Abbruchraten verringern und die Resilienz stärken [4, 5]. Gerade in Zeiten des evidenten Ärztemangels – auch in der Radiologie – fordert die zukünftige Generation ein gutes Ausbildungskonzept, eine aussichtsreiche Karriereperspektive, Zufriedenheit im Job und eine angemessene Work-Life-Balance.

## Was ist Mentoring?

Der Begriff des Mentors kann auf das altgriechische Epos *Odyssee* zurückgeführt werden, in dem Odysseus seinen Sohn Telemachos dem Freund Mentor anvertraut, als er in den Krieg um Troja zieht. Mittlerweile hat sich die Bedeutung des Begriffs weiterentwickelt, und so ist eine umfassende Definition aufgrund der Komplexität nicht einfach zu finden. Mentoring bezeichnet heute allgemein einen länger andauernden Prozess, bei dem ein Mentor durch das Teilen von Erfahrungen und Ansichten seinem Schützling (= *Mentee*) hilft, berufliche wie private Ziele zu erreichen und mögliche Probleme zu überwinden [1, 2, 5].

Es werden verschiedene Arten des Mentorings unterschieden (Tab. 1), wobei die Grenzen zum Teil fließend sind. Grob kann zwischen beteiligten Personen einerseits, beispielsweise Peer-Mentoring, Eins-zu-eins-Mentoring oder Mentoring mit mehreren Mentoren und der Strukturierung des Mentorings andererseits, also formell, informell oder als Hybridform, differenziert werden. Dabei weisen die jeweiligen Modelle eigene Vor- und Nachteile auf, sodass je nach Institution, Größe des Programms, beteiligten Personen und Zielsetzung ausgewählt werden kann.

**Table 1:** 

Art	Beschreibung	Vorteil	Nachteil
Nach Struktur			
Formell	Strukturiertes Mentoring mit schriftlicher Initialvereinbarung, Fragebögen, messbaren Zielen und Erfolgsevaluationen	Progress messbar. Geeignet insbesondere im akademischen Umfeld und zum Vermitteln konkreter Fähigkeiten	Vertraulichkeit ggf. eingeschränkt. Soft Skills oft nicht konkret messbar
Informell	Freie Art des Mentorings. Hier ist eher der begleitete Weg das Ziel als konkrete Fähigkeiten oder das Bearbeiten einer Agenda	Eher Fokus auf Persönliches. Bedarfsgerecht adaptierbar, Inhalt frei wählbar	Wenig objektivierbar. Unterschiedliche Vorstellungen können hier unausgesprochen aufeinandertreffen
Hybrid/strukturiert selbstbestimmt	Hier werden Initialvereinbarung und Fragebögen gestellt, inwiefern diese verwendet werden, bleibt dem Mentoring-Paar überlassen	Sehr individuelle Anwendbarkeit auf Probleme, Fragestellungen und Beteiligte	Eine Erfolgsauswertung ist nur bedingt möglich. Gesamtevaluation ggf. mittels zusätzlicher anonymer Umfrage
Nach beteiligten Personen			
Eins-zu-eins	Das Mentoring findet zwischen einem Mentor und einem Mentee statt	Die Beteiligten können sich aufeinander fokussieren. Insbesondere bei langfristigem Mentoring ratsam	Nicht alle Fragen und Probleme treffen die Expertise des Mentors
Peer-Mentoring	Das Mentoring wird von mehreren Personen der gleichen Stufe untereinander durchgeführt	Häufig geringe Hemmschwelle der Beteiligten. Aktualität der Themen für alle	In der Regel keine langjährige Erfahrung und kein großes Netzwerk
Mehrere Mentoren	Ein Mentee wird von mehreren Mentoren betreut. Insbesondere beim Erlernen einzelner Fähigkeiten und im akademischen Bereich verbreitet	Jeder Mentor kann nach einem speziellen Bedürfnis ausgewählt werden	Oft keine tiefe oder langfristige Mentorenpartnerschaft. Die Kapazitäten für die einzelnen Mentoren sind begrenzt. Persönliches bleibt oft außen vor
Distance Mentoring	Der Mentor ist in einem anderen Institut oder Berufsfeld tätig. Treffen oft virtuell	Verbindungen in spezielle Zentren möglich. Input und Netzwerk können auch außerhalb der Medizin genutzt werden	Eingeschränkte Kommunikationsmöglichkeit im virtuellen Raum. Oft keine niederschwellige Verfügbarkeit

## Kriterien zur Wahl eines Mentors

Wer als Mentor in ein Programm einsteigt, sollte das zu allererst freiwillig tun. Oft bietet es sich an, als Institution einen Pool an freiwilligen Mentoren auszumachen. Nach Möglichkeit deckt dieser ein breites Spektrum an Arbeitsschwerpunkten (klinisch/akademisch), Rängen innerhalb der Abteilung und persönlichem Hintergrund (Herkunft, Familie, Alter, Geschlecht) ab. Zurückhaltend sollten jedoch Instituts- und Abteilungsleiter eingesetzt werden, um eine Überlagerung der Funktionen Vorgesetzter/Mentor und damit möglicherweise einhergehende Befangenheit zu vermeiden. Als wesentliche Erfolgsfaktoren ließen sich die Verfügbarkeit des Mentors und der regelmäßige Kontakt ermitteln [6, 7]. Weitere wichtige Eigenschaften sind Ehrlichkeit und die Fähigkeit, eine offene und nicht voreingenommene Gesprächsatmosphäre zu schaffen, um eine Mentee-orientierte Agenda auszuarbeiten. Aktives Zuhören, Eingehen auf die Bedürfnisse des Mentees und Teilhabe am Erreichen seiner Ziele sind daher wesentlich.

Für den Vorbildcharakter eines Mentors scheinen besonders klinische und didaktische Fähigkeiten ins Gewicht zu fallen [6]; für die praktische Unterstützung des Mentees hingegen ist vielmehr ein ausgeprägtes Netzwerk unter Kollegen entscheidend. Eher sekundär – wenn auch hilfreich – wurden demnach der akademische Rang und aufgabenorientierte Unterstützung, beispielsweise Hilfe bei konkreten klinischen Fällen, Ethikanträgen oder Forschungsförderungen, eingestuft [5]. Diese Aufgaben können oft durch Kollegen und Forschungsgruppenmitglieder abgedeckt werden, ohne dass die persönliche Ebene eines Mentors notwendig ist. Eine genaue Evaluation der eigenen zeitlichen Ressourcen, ob und wie viele Mentees ein Mentor betreuen kann, wird empfohlen.

## Positive Beeinflussung des Mentorings als Mentee

Das Mentoring kann insbesondere auch durch den Mentee mitgestaltet werden. Das Respektieren und Wertschätzen der oft begrenzten Ressourcen des Mentors spielen hier eine wichtige Rolle; eine gute Vorbereitung auf Treffen, ggf. mit Themenagenda ist also zu empfehlen. Ein Mentee-orientiertes Konzept bedeutet auch, dass der/die zu Betreuende proaktiv Themen setzt, Erwartungen anspricht und Ziele formuliert. Falls regelmäßige Treffen nicht von der Institution vorgegeben werden, steht durchaus auch der Mentee in der Verantwortung, hier die Initiative zu ergreifen. Nicht immer werden die Meinungen von Mentor und Mentee deckungsgleich sein; auch nach ausführlicher Diskussion können divergierende Meinungen verbleiben. Diese gilt es dann zu akzeptieren und sich ggf. darauf zu einigen, sich nicht einig zu sein. Trotz der größten Erfahrung wird kein Mentor alle Fragen beantworten oder alle Probleme lösen können. Letztendlich bezieht sich die Aufgabe eines Mentors hier eher auf Hilfe beim Suchen eines eigenen Lösungswegs, ggf. durch Einbringen von Erfahrungen oder das Aktivieren eines Netzwerks.

Problematisch wird die Interaktion innerhalb eines Mentoring-Paares insbesondere bei fehlendem Engagement oder Greifbarkeit des Mentors. Umfragen zeigen, dass dieser Punkt von den Mentoren regelmäßig nicht erkannt wird [7], daher hilft oft schon das direkte Ansprechen. Sollte sich keine Besserung einstellen oder sollten gar persönliche Differenzen vorliegen, kann ggf. ein weiterer Mentor gesucht werden, auf den man seine Kapazitäten konzentriert. Auch das Beenden einer Mentorenschaft oder ein Mentorwechsel sind prinzipiell legitim, gehen manchmal jedoch mit berufspolitischen Risiken einher, sodass ein feinfühliger Umgang mit der Situation und eine genaue Abwägung im Einzelfall von Nöten sind.

## Nutzen für Mentee und Mentor

Die Vorteile des Mentorings für den Mentee sind zahlreich: Einerseits erhält der Mentee Zugriff auf Erfahrungen und Ansichten einer erfahrenen Radiologin oder eines erfahrenen Radiologen, die insbesondere die oft vulnerable Phase des Berufseinstiegs begleiten können. Doch auch darüber hinaus kann ein Mentor Erfahrungen zur beruflichen Weiterentwicklung, Subspezialisierung oder Neuorientierung teilen und durch Integration in sein Netzwerk politische und akademische Projekte vorantreiben. Andererseits werden im Mentoring oft auch Probleme bezüglich der Vereinbarkeit von Familie und Beruf, Umgang mit einschneidenden privaten Lebenssituationen oder Probleme mit Kollegen thematisiert [3–5]. Durch Reflexion, neue Perspektiven und Einbringen persönlicher Erfahrungen des Mentors wird der Umgang mit schwierigen Situationen erleichtert und die Resilienz des Mentees gesteigert.

Auch wenn der Mentor primär eine nährende Rolle einnimmt, sehen sich viele von ihnen selbst auch als Profiteure des Programms: Der direkte Draht zu den jüngeren Kollegen bringt den Zugang zu neuen Ansichten und damit auch eine Möglichkeit zu reflektieren und seine eigenen Ziele zu hinterfragen, zu erkennen und neu zu definieren. Begleitet von Weiterbildungen wird die Entwicklung von Soft Skills und Führungskompetenz gefördert [4, 5]. Auch konkrete technische Fähigkeiten werden oft von der Generation der „digital natives“ weitergegeben. Nicht zuletzt geht es vielen jedoch primär darum zu helfen, im Sinne eines Generationenvertrags etwas zurückgeben zu können und die Begeisterung an der Radiologie auch für sich selbst neu zu entdecken.

## Institutionelle Rahmenbedingungen

Der Erfolg eines Mentoring-Programms hängt entscheidend auch von den institutionellen Rahmenbedingungen ab. Zunächst ist eine klare Unterstützung der Klinik, Abteilung oder Praxis nötig. Das angestrebte Format wird idealerweise von den Mitarbeitern selbst definiert; hierdurch werden eine größere Akzeptanz und eine stärkere Beteiligung erzielt [4]. Aufgaben, Ziele und Umfang des Mentorings können in einem frei zugänglichen Dokument nachgeschlagen werden. Für die Leitung, die Evaluation und den reibungslosen Ablauf des Programms sollte die Institution sorgen. Zu Beginn ist es hilfreich, einen Pool an freiwilligen Mentoren zu identifizieren, aus denen die Mentees wählen können. Eine Limitierung auf ein bis zwei Mentees pro Mentor ist sinnvoll, um ausreichend Kapazitäten zu gewährleisten.

Für die Treffen zwischen Mentor und Mentee ist Vertraulichkeit unabdingbar. Daher bleibt ein wesentlicher Diskussionspunkt, ob und inwiefern die Gespräche dokumentiert werden sollen. Gute Argumente lassen sich hier für verschiedene Ansätze finden: Im Rahmen des formellen Mentoring (Tab. 1) hat sich das Festhalten von Problemen, Lösungsansätzen und konkreten Zielen durchgesetzt, sodass hier ein Progress messbar ist. Die Ziele können dabei nach den SMART-Kriterien (spezifisch, messbar, achievable – also erreichbar, reasonable – also angemessen und mit Time-Line) angelegt werden. Argumente, die eher gegen eine Dokumentation sprechen, beziehen sich oft auf die Vertraulichkeit und die eingeschränkte Messbarkeit einzelner Endpunkte, beispielsweise „gute Patientenkommunikation“ oder „eine gute Radiologin/ein guter Radiologe sein“.

Unumstritten ist hingegen die Relevanz regelmäßiger Treffen (meist halb- oder vierteljährlich), die durch vorgegebene Termine und Erinnerungsmails sowie ungestörte Zeitslots unterstützt werden können. Diese Treffen geben eine Basis, die bedarfsgerecht erweitert werden kann. Gemeinsame Aktivitäten mehrerer Mentoring-Paare helfen zudem, das Programm zu beleben. Weiterbildungen geben den Mentoren das entsprechende Rüstzeug an die Hand, ihre Aufgaben zu erfüllen und ihre Kompetenzen auszubauen. Insbesondere in persönlichen Belastungssituationen und bei schwerwiegenderen Problemen am Arbeitsplatz kann eine Besprechung unter den Mentoren ein hilfreicher Ansatz sein. In einzelnen Fällen werden die Mentoren jedoch an die Grenzen ihrer Kompetenzen und Kapazitäten stoßen, sodass eine Anbindung an den psychosozialen Dienst oder ein Kriseninterventionsteam angeraten ist, auch wenn es nur selten zu einer solchen Eskalation kommt.

## Organisationseinheiten des Mentorings

Die Vorteile eines Mentoring-Programms beschränken sich nicht nur auf eine Abteilung oder Praxis, sondern können auch interdisziplinär innerhalb eines Krankenhauses Vorteile bieten, insbesondere wenn es um Forschungsprojekte oder interdisziplinäre Zusammenarbeit geht (beispielsweise zwischen MSK-Radiologie und Orthopädie oder interventioneller Radiologie und Hepatologie). Zudem werden auch überregionale Mentoring-Programme innerhalb der Radiologie angeboten, die einen Wissenstransfer von spezialisierten Zentren sowie die wissenschaftliche Zusammenarbeit und Networking erleichtern sollen. Des Weiteren werden an vielen Universitäten Mentoring-Programme bereits für Studierende angeboten, an denen das Interesse für Radiologie geweckt werden kann.

## Fazit

In Zeiten von Ärztemangel und Arbeitsverdichtung bietet Mentoring eine effektive Methode, um Resilienz und Job-Zufriedenheit zu steigern sowie Abbruchraten, Stress und Burnout zu vermindern. Dabei bieten Mentoring-Programme nicht nur für Mentees den Vorteil, von Erfahrung, Anleitung und einem bestehenden Netzwerk zu profitieren. Auch Mentoren können einen Nutzen durch die Entwicklung von Soft Skills, Führungskompetenz und neue Ansichten ziehen.

Als zentrale Eigenschaften eines guten Mentors werden eine niederschwellige Verfügbarkeit, Unvoreingenommenheit und Offenheit für ein Mentee-orientiertes Vorgehen angesehen. Den Vorbildcharakter erhält der Mentor dabei primär durch gute klinische und didaktische Fähigkeiten. Der Mentee kann die Verbindung positiv beeinflussen, indem er proaktiv handelt und die Ressourcen des Mentors respektiert. Für den Erfolg eines Mentoring-Programms ist eine aktive und supportive Rolle der organisierenden Institution unabdingbar. Insgesamt kann somit durch ein Mentoring-Programm mit überschaubarem Aufwand der Berufsalltag für junge Radiologen immens verbessert werden.
